# The mRNA capping enzyme of *Saccharomyces cerevisiae* has dual specificity to interact with CTD of RNA Polymerase II

**DOI:** 10.1038/srep31294

**Published:** 2016-08-09

**Authors:** Akhilendra Pratap Bharati, Neha Singh, Vikash Kumar, Md. Kashif, Amit Kumar Singh, Priyanka Singh, Sudhir Kumar Singh, Mohammad Imran Siddiqi, Timir Tripathi, Md. Sohail Akhtar

**Affiliations:** 1 Molecular and Structural Biology Division, CSIR-Central Drug Research Institute, Sector 10, Jankipuram Extension, Lucknow, PIN 226 031, India; 2 Molecular and Structural Biophysics Laboratory, Department of Biochemistry, North-Eastern Hill University, Shillong, PIN 793022, India

## Abstract

RNA Polymerase II (RNAPII) uniquely possesses an extended carboxy terminal domain (CTD) on its largest subunit, Rpb1, comprising a repetitive Tyr_1_Ser_2_Pro_3_Thr_4_ Ser_5_Pro_6_Ser_7_ motif with potential phosphorylation sites. The phosphorylation of the CTD serves as a signal for the binding of various transcription regulators for mRNA biogenesis including the mRNA capping complex. In eukaryotes, the 5 prime capping of the nascent transcript is the first detectable mRNA processing event, and is crucial for the productive transcript elongation. The binding of capping enzyme, RNA guanylyltransferases to the transcribing RNAPII is known to be primarily facilitated by the CTD, phosphorylated at Ser_5_ (Ser_5P_). Here we report that the *Saccharomyces cerevesiae* RNA guanylyltransferase (Ceg1) has dual specificity and interacts not only with Ser_5P_ but also with Ser_7P_ of the CTD. The Ser_7_ of CTD is essential for the unconditional growth and efficient priming of the mRNA capping complex. The Arg159 and Arg185 of Ceg1 are the key residues that interact with the Ser_5P_, while the Lys175 with Ser_7P_ of CTD. These interactions appear to be in a specific pattern of Ser_5P_Ser_7P_Ser_5P_ in a tri-heptad CTD (YSPTS_P_PS YSPTSPS_P_ YSPTS_P_PS) and provide molecular insights into the Ceg1-CTD interaction for mRNA transcription.

Eukaryotic mRNAs are transcribed by RNA polymerase II (RNAPII) and the pre-mRNAs undergo several processing events such as 5 prime (5′) capping, splicing, polyadenylation etc. before becoming the mature mRNA. The 7-methyl guanosine (m7G) capping by the RNA guanylyltransferase is the first co-transcriptional modification of mRNA occurs, when the transcript is only 25–30 nucleotides long. The 5′ capping helps preventing mRNA decay and play a distinct role during the mRNA biogenesis^1^,^2^.

In *Saccharomyces cerevisiae*, the heterodimeric capping enzyme complex, RNA guanylyltransferase (Ceg1) and RNA triphosphatase (Cet1) are directly recruited to the carboxy-terminal domain (CTD) of Rpb1, the largest subunit of RNAPII^3^,^4^,^5^. The CTD is a highly conserved unusual domain consisting of repeating heptapeptide consensus sequence (Tyr_1_Ser_2_Pro_3_Thr_4_Ser_5_Pro_6_Ser_7_), whose copy number generally varies with the organism complexity, such as protozoa containing 15, budding yeast 26, and humans 52 repeats^1^,^2^. The functional unit of CTD is di-heptapeptide and minimum 11 repeats are required for the unconditional viability in budding yeast^6^,^7^. The CTD was primarily known to be phosphorylated at Ser_2_ (Ser_2P_) and Ser_5_ (Ser_5P_). These two phosphorylations were considered essential for recruiting factors important for the activity of RNAPII during mRNA biogenesis^1^,^2^. Of late the Ser_7_ phosphorylation was also identified during the transcription of snRNA and mRNA. Although its function is obscure in budding yeast, it contributes to the expression of noncoding RNA and mRNA splicing in mammalian cells^1^,^8^,^9^,^10^,^11^. The phosphorylation of Ser_7_ is dependent on Ser_5_ phosphorylation however, the Ser_2_ and Ser_5_ phosphorylation is not dependent on Ser_7_ phosphorylation^8^.

The CTD may undergo dynamic and combinatorial epigenetic phosphorylations (Ser_2_, Ser_5_ and Ser_7_) with its minimal presence or enrichment at given position of the gene. The “CTD code” hypothesizes that the sequential modifications of the CTD marks specify a recognition code similar to the histone code^1^. A distinct pattern of all the serine phosphorylations is observed in the protein-coding genes and the role for Ser_7P_ in mRNA transcription is consistent with the observation that highly transcribed genes show high levels of this mark^2^. The actual phosphorylation pattern and sequence in CTD, which promote protein binding or dissociation or regulate their function remains unknown *in vivo.* Furthermore, the studies on the CTD phosphorylation and function majorly relied on the use of commercial antibodies whose validity remains a subject of great debate^9^. Thus we are dealing with a situation that hinders the in depth understanding of the properties and function of CTD *in vivo.*

The phosphorylation of Ser_5_ of CTD by Kin28/Cdk7 results in the coordinated recruitment of the mRNA capping complex consists of Ceg1-Cet1^4^,^5^. The physical interaction between the Ser_5P_ of CTD and Ceg1 is suggested to be required for the efficient formation of 5′ m7G-capping of mRNA^12^,^13^. The RNA guanylyltransferases are conserved throughout the evolution and contain two domains, a nucleotidyl transferase (NT) domain and a C-terminal oligonucleotide binding (OB) domain^4^,^14^. The phosphorylated CTD interacts directly with the NT domain of Ceg1, but facilitates guanylation only in the presence of Cet1^3^,^4^,^15^. The crystal structure of *Candida albicans* RNA guanylyltransferase (Cgt1) bound to a 17 amino acid CTD phosphopeptide shows a saddle shaped CTD binding surface containing CTD docking sites (CDS) or pockets where Ser_5P_ is anchored. The Lys152, Arg157 and Tyr165 of CDS1 observed to interact with Ser_5P_ through electrostatic and hydrogen bond interaction, whereas the Arg140, Lys178 and Lys193 interact from CDS2 and make similar interactions^14^. The CTD content is similar in the budding yeast and bimorphic fungus *Candida albicans.* The complementation experiment, where the physiological role of individual mutations of probable CDS residues suggests that no single amino acid is essential for *ceg1Δ* cell growth. However, R157 of Cgt1 has shown a detrimental growth defect^14^.

Here we report the dual specificity of Ceg1, which not only interacts with Ser_5P_ but also with Ser_7P_ of CTD. We also report the residues of Ceg1 and their nature of interaction with Ser_5P_ and Ser_7P_ of CTD. The interaction between CTD and Ceg1 is appears to be in a Ser_5_Ser_7_Ser_5_ manner in order to facilitate an efficient mRNA capping.

## Results

### Ceg1 interacts with both Ser_5P_ and Ser_7P_ of CTD

**Figure 1:**
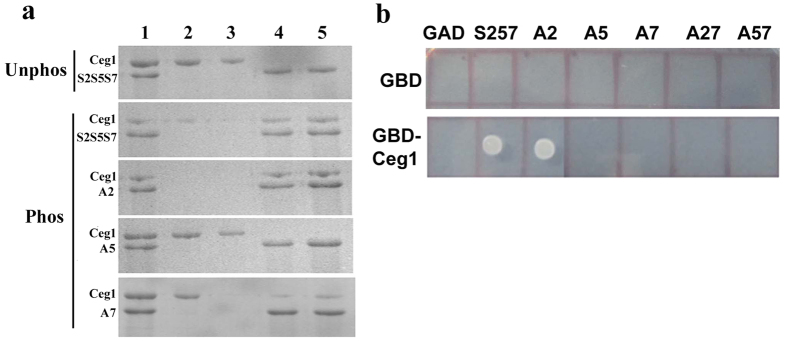
Ceg1 interacts with the phosphorylated Ser_5_ and Ser_7_ of CTD. (**a**) SDS PAGE analysis of the pull down assay where Ceg1 does not bind to the unphosphorylated CTD, but binds to the CTD phosphorylated by Kin28. In the first panel, lane 1 represents the purified Ceg1 (5 μg) incubated with an equal amount of unphosphorylated CTD, lane 2 and 3 represents the washed samples and Lane 4 and 5 represents the samples eluted after the extensive wash of the complex. The subsequent panel (top to bottom) represents pull down complex for CTD phosphorylated at all the three serines (S2S5S7), S5S7, S2 and S2S5 respectively. (**b**) Yeast two hybrid assays, where GAD vector (pGADCU-1) alone or the GAD vector expressing either the consensus CTD with all the three Ser residues (Ser2Ser5Ser7 or S257) or Ser mutants (Ser_2_ mutant [A2] or Ser_5_ mutant [A5] or Ser_7_ mutant [A7] or Ser_2_ + Ser_7_ double mutant [A27] or Ser_5_ + Ser_7_ double mutant [A57]) was co-transformed into pJ69-4A strain either with GBD vector (pGBDCU-1) alone or GBD-Ceg1. The profiles show the growth due to the transcription of a HIS3 reporter, resulting from the interaction between two-hybrid fusion proteins.

The phosphorylation of Ser_5_, primarily by the TFIIH-associated kinase Kin28, enhances the association of CTD with the m7G mRNA capping machinery^16^,^17^. However, Kin28 also phosphorylates Ser_7_ of CTD (only on the prephosphorylated Ser_5_ heptad) and the role of this phosphorylation in mRNA transcription remains obscure^2^,^8^,^9^. Since Kin28 marks both, the Ser_5_ and Ser_7_ phosphorylation at the 5′ end of the gene and also the occupancy profile of Ser_5P_ and Ser_7P_ overlaps in most of the cases in this region, the role of dual phosphorylation of CTD either in Ceg1 recruitment and subsequent role in mRNA capping cannot be ruled out. In a pull down assay, an interaction between Ceg1 and the CTD phosphorylated at Ser_5_ and Ser_7_ (by Kin28) was observed (Fig. 1a). The Ceg1 did not show any interaction with unphosphorylated CTD (CTD-unphos) or CTD phosphorylated only at Ser_2_ (A5) and was washed away before the elution (Fig. 1a, first and fourth panel). However, an interaction between Ceg1 and CTD was observed in cases where either all the three serines or at least two serines (Ser_5_ and Ser_7_) were phosphorylated (Fig. 1a, second and third panel). A compromise in the binding of Ceg1 with CTD was observed in cases where only Ser_7_ was mutated (Fig. 1a, fifth panel). The compromise in the efficient binding between Ceg1 and CTD in the absence of Ser_7P_ suggests that a specific and more prominent interaction exists between the Ceg1 and Ser_5P_ (Fig. 1a, fifth panel).

We subsequently verified the interaction of Ceg1 with Ser_5P_ and Ser_7P_ of CTD by carrying out a modified yeast two hybrid (Y2H) analysis, where a phoshorylation dependent binding of Ceg1 to the CTD was observed^17^. The binding of Ceg1 to the consensus CTD (having all the three serines) or CTD with single or double point mutations for Ser was analyzed by Y2H (Fig. 1b). The cells expressing GBD-Ceg1 (Ceg1 cloned downstream to Gal4 binding domain) grew on medium lacking Ura, Leu and His, when co-expressed with GAD-CTD (CTD cloned downstream to Gal4 activation domain) containing either all the three conserved serines in a heptad (Ser2Ser5Ser7 or S2S5S7 or S257) or at least have the Ser_5_ and Ser_7_ conserved (A2). On similar experimental condition the absence of growth in case of CTD mutants (A5, A7, A27 and A57) indicates the lack of significant interactions between GBD-Ceg1 and GAD-CTDs for the reporter gene expression. The absence of reporter gene expression, especially in the case of A7, clearly suggests an important role of Ser_7_ in the binding of mRNA capping enzyme to CTD.

Since Ceg1 is already known to interact with Ser_5P_ of CTD for mRNA capping, its further interaction with Ser_7_ appears as a stabilizer or a place keeper to increase the specificity of the interaction. This is supported by the fact that the mutation of Thr_4_ and Ser_7_ residues of the budding yeast (by replacing the CTD of budding yeast with that of *Mastigamoeba invertens* which contains 25 heptads of YSPASPA) shows compromise in the growth^18^.

### Ser7 mutation affects mRNA transcription

**Figure 2:**
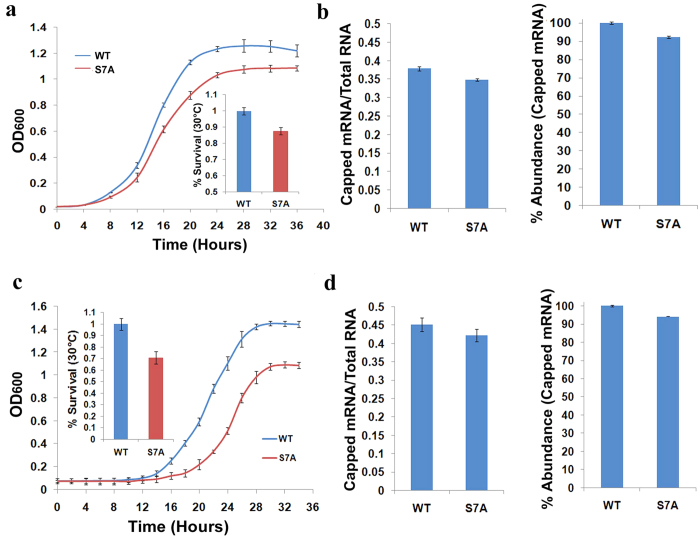
Ser7 mutation affects the unconditional growth and reduces the mRNA transcript capping. (**a**) Growth curves and the relative change in the growth for *S. cerevisiae* containing consensus (WT) and mutatated (S7A) CTD heptad at 30 °C. (**b**) S7A reduces mRNA transcript capping. The fractional decrease in capped mRNA as well as its relative abundance was measured by doing immunoprecipitation experiment using five micrograms of RNA and H20, an anti-5′ cap monoclonal antibody. The standard deviations are displayed as error bars. (**c**) Growth curves and the relative change in the growth for *S. pombe* containing full length consensus (WT) and mutated (S7A) CTD heptad at 30 °C. (**d**) S7A reduces mRNA transcript capping. The fractional decrease in capped mRNA as well as its relative abundance in S7A as mentioned above.

The phosphorylation of promoter bound RNAPII-CTD by Kin28 is thought to play a critical role in the transcription initiation, promoter clearance and enhancing 5′ capping of the nascent transcripts^1^,^2^. The chemical inhibition of the analog sensitive Kin28 (Kin28-as) leads to the reduction in 5′ capping of transcripts and steady state mRNA levels^14^. However, these events were primarily thought of the consequence of the reduction in the Ser_5P_ of CTD near promoter of the gene. To see the effect of the role of Ser_7_ phosphorylation in mRNA transcription, we constructed CTD mutant where the Ser_7_ was substituted for Ala (RNAPII-CTD-Ser_7_Ala or S7A). The mutation of Ser_7_ to Ala, decreased the growth of strain relative to the wild type and suggests that this mutation does not support the unconditional growth and have a role in gene regulation (Fig. 2a). To check the effect of Ser_7_ phosphorylation on 5′ mRNA capping, the capped mRNA transcripts from the budding yeast strain containing consensus (WT) or mutated Ser_7_ (S7A) were immunoprecipitated and the level of m^7^G capping was quantified using H20, an anti-5′ cap monoclonal antibody (Fig. 2b). To affirm the observation, we also checked the effect of Ser_7_ mutation in fission yeast with mutated S7A construct^19^. Here too, the S7A affects the growth and mRNA capping similar to that observed in the budding yeast (Fig. 2c,d). The above studies affirm the role of Ser_7_ phosphorylation as a place keeper to help efficient priming of 5′ mRNA capping complex in yeast.

### Arg159 and Arg185 of Ceg1 interacts strongly with CTD

**Figure 3:**
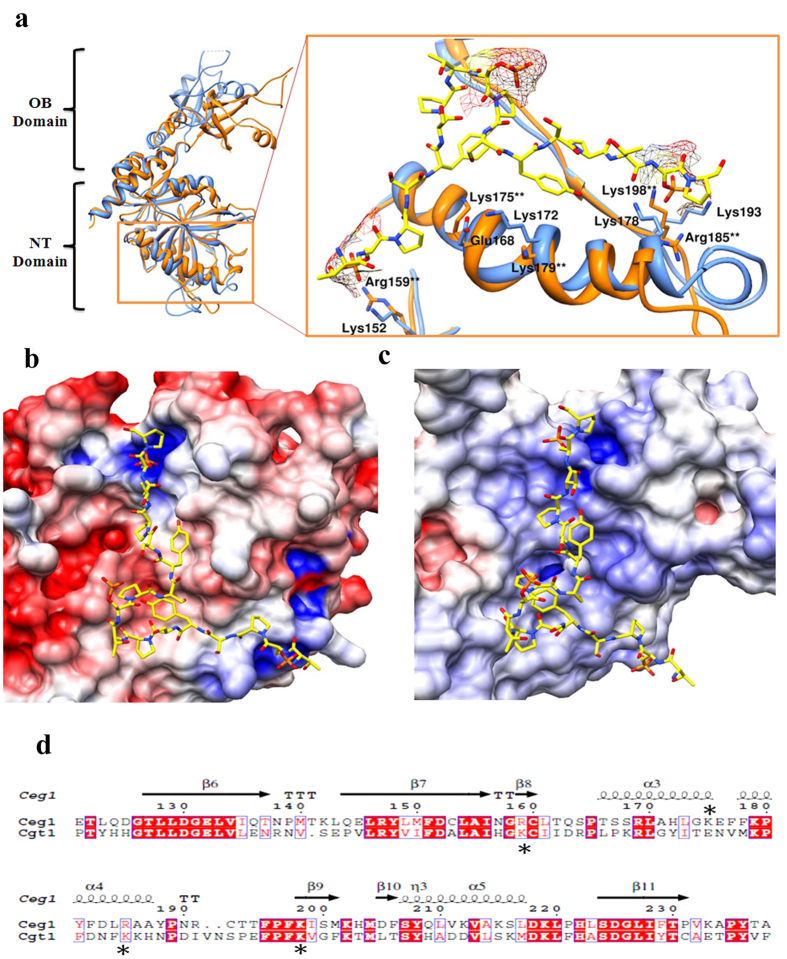
Structural superimposition shows key residues of Ceg1 interact with the phosphorylated CTD. (**a**) The superimposition of the crystal structure of Cgt1 (sky blue) and Ceg1 (orange) with an enlarged region, shows the CTD binding region where **indicates the residues from Ceg1 interacting with phosphorylated CTD. (**b**) The surface electrostatic potential of the NT domain complex of Cgt1 with phosphorylated CTD sequence show clustered positive patches in the form of two pockets **(c)** The surface electrostatic potential of the NT domain complex of Ceg1 with phosphorylated CTD sequence however shows dispersed and clustered positive patches. (**d**) Sequence alignment of the residues of NT domain of Ceg1 and Cgt1. Residues in the red background are fully conserved and residues in red colour are semi conserved. Symbol (*) represents the location where mutations were carried out.

To analyze the residues involved in the interaction between Ceg1 and phosphorylated CTD, we aligned the conserved nucleotidyl transferase (NT) domain of Ceg1 with the co-crystal structure of Cgt1 bound with a 17 amino acid of CTD sequence (TS_P_PSYSPTS_P_PSYSPTS_P_P) phosphorylated at Ser_5_ on each heptad using UCSF Chimera (Fig. 3a). We carried out the structural alignment and observed that, both the proteins exhibit a similar structural pattern, but relatively different surface electrostatic potential (Fig. 3b,c). Contrary to clustered positive patches in Cgt1, both dispersed and clustered positive patches are present in Ceg1, depicting a different binding properties for the phosphorylated CTD in *S. cerevisiae*. The residues Arg159, Arg185 and Lys198 of Ceg1 were observed to make direct contacts with Ser_5P_ (at position 2 and 16 of the peptide) in the superimposed structure model. These three residues are also conserved in Cgt1 (Fig. 3d). Out of two other electropositive residues (Lys175 and Lys179), which appears to make contact with CTD peptide, Lys175 is in close proximity to Ser_7_ (at position 11) of second heptad (Fig. 3a).

**Figure 4:**
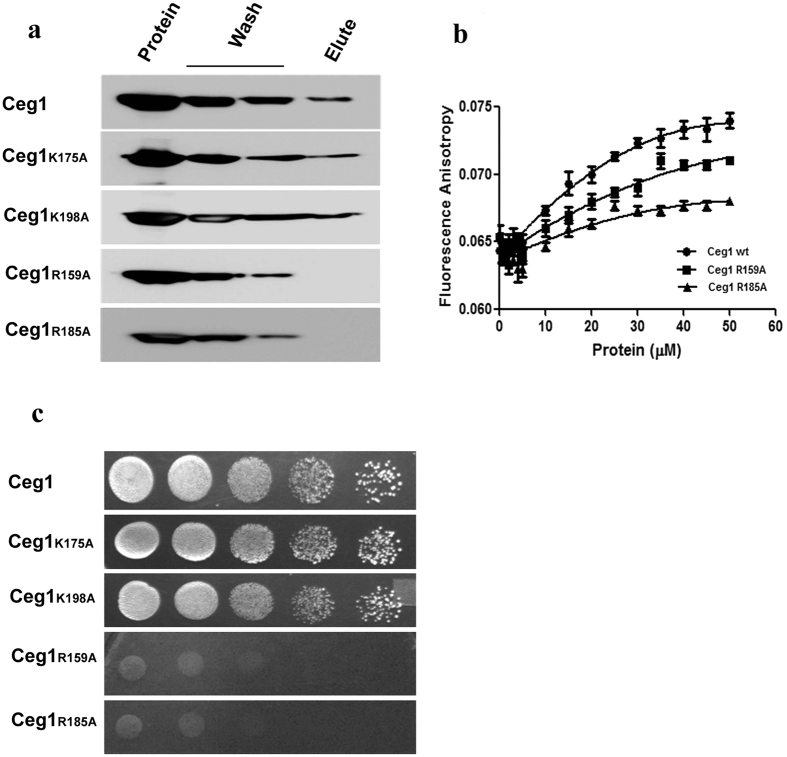
Arg159 and Arg185 of Ceg1 makes strong interaction with Ser_5P_ of CTD. (**a**) Immunodetection (anti-his) of the pull down sample where his-tagged Ceg1 or Ceg1 mutants (first lane) were incubated with CTD (14 repeats) phosphorylated by the Kin28 for their possible interaction. The samples were washed (two middle lanes) and subsequently the eluents were analyzed for the presence of Ceg1 or its mutants bound to the phosphorylated CTD (last lane). (**b**) The fluorescence anisotropy measurements, where 2 μM of FAM-CTD-Ser_5P_ peptide was titrated against increasing concentrations of Ceg1 (•), Ceg1_R159A_ (■) and Ceg1_R185A_ (▲) respectively to find out their binding efficiency. (**c**) Yeast two hybrid assays, where GAD-CTD was co-transformed into pJ69-4A strain either with GBD-Ceg1 or with Ceg1 mutants (GBD-Ceg1_K175A_ or GBD-Ceg1_K198A_ or GBD-Ceg1_R159A_ or GBD-Ceg1_R185A_). The profiles show the growth due to the transcription of a HIS3 reporter, resulting from the interaction between two-hybrid fusion proteins.

To the residues of Ceg1, observed to make a possible interaction with CTD, a point mutation was created for Arg159 (Ceg1_R159A_), Lys175 (Ceg1_K175A_), Arg185 (Ceg1_R185A_) and Lys198 (Ceg1_K198A_) and the pull down assay was carried out at similar condition as described above with CTD phosphorylated by Kin28 (Fig. 4a). The mutants Ceg1_R159A_ and Ceg1_R185A_ lost interaction with CTD and was washed away before elution. However, Ceg1_K175A_ and Ceg1_K198A_ did not lose interaction with CTD and were detected in the eluent. We further checked the binding efficiency of Ceg1_R159A_ and Ceg1_R185A_ with commercial CTD peptide phosphorylated at Ser_5_ by doing fluorescence anisotropy assay (Fig. 4b). The titration of phospho peptide (YSPTS_P_PS-YSPTS_P_PS-YSPTS_P_PS) with increasing concentrations of protein shows preferential binding of Ceg1 to Ser_5P_ as compared to the mutants Ceg1_R159A_ and Ceg1_R185A_. The observed K_d_ for Ceg1, Ceg1_R159A_ and Ceg1_R185A_ were ~460.7 μM, ~2373 μM and ~2285 μM respectively suggests that the residues Arg159 and Arg 185 of Ceg1 makes a significant interaction with Ser_5P_ of CTD.

To confirm the *in vivo* efficiency of Ceg1 and its mutants in binding to the CTD, a yeast two hybrid analysis was carried out as described above (Fig. 4c). We checked the binding of GBD-Ceg1, GBD-Ceg1_R159A_, GBD-Ceg1_K175A_, GBD-Ceg1_R185A_ and Ceg1_K198A_ with the consensus GAD-CTD fusion *in vivo*. The cells expressing GBD-Ceg1, GBD-Ceg1_K175A_ and GBD-Ceg1_K198A_ grew on medium lacking Ura, Leu and His, when co-expressed with GAD-CTD. However, cells expressing GBD-Ceg1_R159A_, and GBD-Ceg1_R185A_ did not grow optimally on similar media indicating the lack of significant interactions with GAD-CTD *in vivo.*

The above studies suggest a strong interaction between the Ser_5P_ of CTD and Arg159 and Arg185 of Ceg1. Since, the interaction of Ceg1 to phosphorylated CTD is primarily determined by phosphorylated Ser5, the supportive interaction with Lys175 of Ceg1 was not expected to completely block the interaction between pCTD and Ceg1. The strong interaction is provided by the arginine residue in both CDS1 and CDS2. In CDS1, Arg159 provides the major stabilizing interaction, which came out as crucial residue in our *in vitro* as well as *in silico* studies. In CDS2, along with K198, Arg147 and Arg185 are two important residues, which interact strongly with phosphorylated serine. Due to presence of Arg147 and Arg185, role of K198 in CTD binding appears negligible. In the fluorescence competitive assay, the Kd for Ceg1 or Ceg1_K175A_ or Ceg1_R198A_ was also almost same.

### MD simulations reveal a pattern of interaction between Ceg1 and CTD

The structure of CTD is very flexible and can adopt multiple conformations. The dynamic phosphorylation patterns of CTD in the transcription cycle undergo significant changes from initiation to termination, however the exact phosphorylation pattern *in vivo* remains unknown till date^2^,^9^. It was reported that there may be only a single phosphorylation per heptad repeat (YSPTSPS), however few recent studies suggests a coexistence of Ser_2_ and Ser_7_ phosphorylation on the same heptad repeat^9^. It is known that the Ser_5_ phosphorylation by Kin28 primes the phosphorylation of Ser7, and hence there is almost a negligible possibility of the coexistence of Ser_5P_ and Ser_7P_ on the same heptad repeat *in vivo*^10^,^20^. It is very likely that these two phosphorylation marks are on the two different heptad repeats. It is also supported by the study, where an interaction between Ser_5P_ of different heptad (position 2^nd^ and 16^th^) and Cgt1 was observed in the co-crystal structure of Cgt1 bound with the T**S**_**P**_PS-YSPTS_P_PS-YSPT**S**_**P**_P. In the co-crystal structure, the Ser_7_ of the middle repeat (position 11) appears to be accessible to the binding by Lys175. Furthermore, the complementation experiment shows the compromised growth in case of R157 of Cgt1 (the corresponding amino acid in *S. cerevisiae* is R159) or for the double and triple point mutations in the residues from CDS1 and CDS2^14^. The above observations hint a possible pattern of binding between the residues of the mRNA capping enzyme (from different CDS) and phosphorylated CTD.

**Figure 5:**
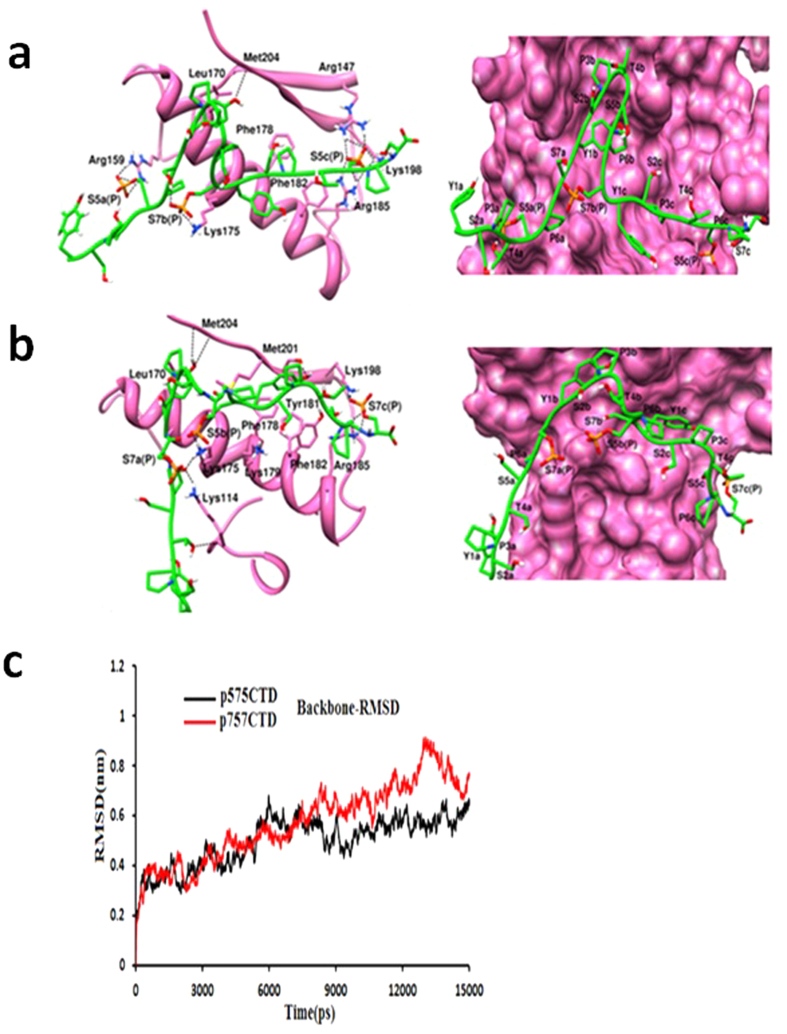
Phosphorylated CTD heptad shows a specific interaction pattern with Ceg1. (**a**) The left panel shows the interaction profile of CTD1 (YSPTS_P_PS-YSPTSPS_P_-YSPTS_P_PS) with Ceg1-CID, obtained after 15 ns MD simulation. The right panel shows the surface view of Ceg1-CID (hot pink) with CTD1. H-bonds are shown in black dotted line. For clear representation only polar hydrogens are shown. (**b**) The right panel shows the interaction profile of CTD2 (YSPTSPS_P_-YSPTS_P_PS-YSPTSPS_P_) with Ceg1-CID, obtained after 15 ns MD simulation. The right panel shows the surface view of Ceg1-CID (hot pink) with CTD2. (**c**) Root mean square deviation of the backbone atoms of Ceg1-CTD1 (black) and Ceg1-CTD2 (red) complexes.

**Figure 6:**
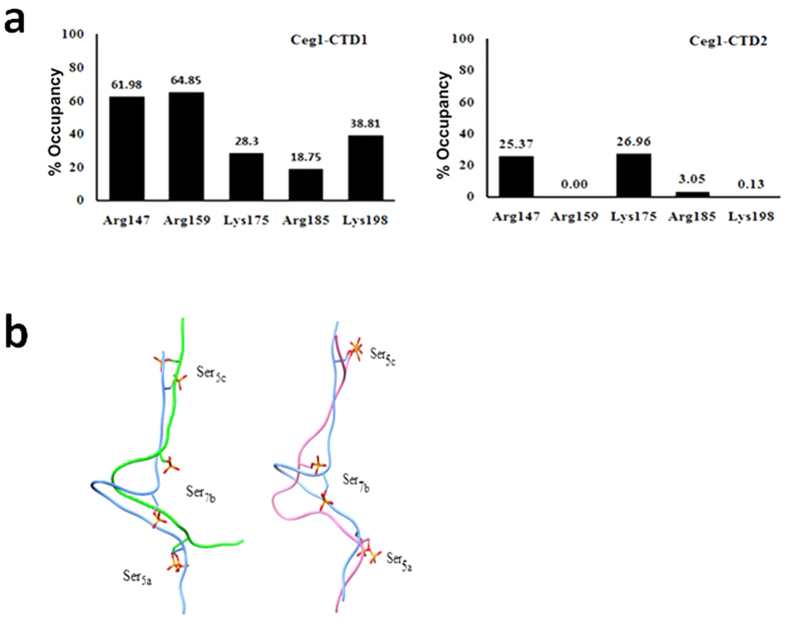
Ceg1 interacts more strongly with CTD1. (**a**) H-bond occupancy plot during 15ns of MD simulation for Ceg1-CTD1 (left panel) and Ceg1-CTD2 (right panel). (**b**) The left panel shows the superimposed CTD1 conformations in case of Ceg1 (blue) and Ceg1_R185A_ (green) after 15ns of MD simulation. The right panel shows the superimposed CTD1 conformation in case of Ceg1 (blue) and Ceg_K198A_ (pink) at similar condition.

In order to understand the structural aspects of the Ceg1-CTD interaction, MD simulation studies were carried out. We used the available Cgt1-CTD co-crystal structure to generate the 3D-models of CTD with different phosphorylation patterns. We first extracted the coordinates of 17 amino acid long phosphorylated CTD from the Cgt1-CTD crystal structure and missing residues at the N-terminal and C-terminal were further added. After generation of 21 amino acid long three CTD heptads (three YSPTSPS motif is termed here as heptad a, b and c respectively), we carried out *in silico* phosphorylation of Ser residues in a 5a7b5c (YSPTS_P_PS-YSPTSPS_P_-YSPTS_P_PS or CTD1) and 7a5b7c (YSPTSPS_P_-YSPTS_P_PS-YSPTSPS_P_ or CTD2) manner. The Ceg1-CTD complexes were subsequently subjected to initial 15 ns MD simulation. The MD studies with the modeled CTD1 and CTD2 were used to get a structural and dynamic view of the Ceg1-CTD interaction as well as the conformational plasticity of the CTD. Simulation result suggests that both the CTD and CID (CTD interacting domain) exhibits an induced fit mechanism to maximize the interaction. Here CTD1 showed strong association with the Ceg1 (Fig. 5a). Residues of Ceg1 which showed interaction with CTD1 were Arg159, Arg147, Lys175, Arg185 and Arg198. The Arg159 is located in the CDS1 and is the only positively charged residue which showed interaction with the Ser_5P_ of the first heptad (a). The Arg147, Lys198 and Arg185 are located in CDS2 and showed the H- bond and electrostatic interactions with Ser_5_ of the third heptad (c). In case of Cgt1, Ser_5P_ makes extensive interaction with the two flanking sites of CID (CDS1 and CDS2), but not with the middle (14). However, in Ceg1, we see that Ser_7P_ can interact with the Lys175 residue. The presence of Lys175 residue makes the middle region of CID of Ceg1 more electropositive than the Cgt1. CTD2 also showed interaction with the Ceg1 (Fig. 5b). The Ser_7_ of first heptad (a) and Ser_5_ of second heptad (b) showed interaction with Lys175. None of the residues of CTD2 showed an interaction with crucial Arg159. This pattern of binding is not supported by our above mentioned *in vitro* and *in vivo* data. Backbone RMSD trajectory for Ceg1-CTD2 shows that the conformation of CTD2 is not stabilized even after 15 ns of simulation. This conformation showed higher RMSD than the Ceg1-CTD1 complex (Fig. 5c). The comparative H-bond occupancy analysis of the complexes also suggest that the Ceg1 interacts more strongly with the CTD1 than CTD2 (Fig. 6). The side chains of Arg159 showed the strong H-bond with the phosphorylated Ser5a of CTD1, while this interaction was absent in case of CTD2. The above results suggest an interaction between Ceg1 and CTD1 with more specificity for Ser5aSer7bSer5c (S5S7S5) manner.

**Figure 7:**
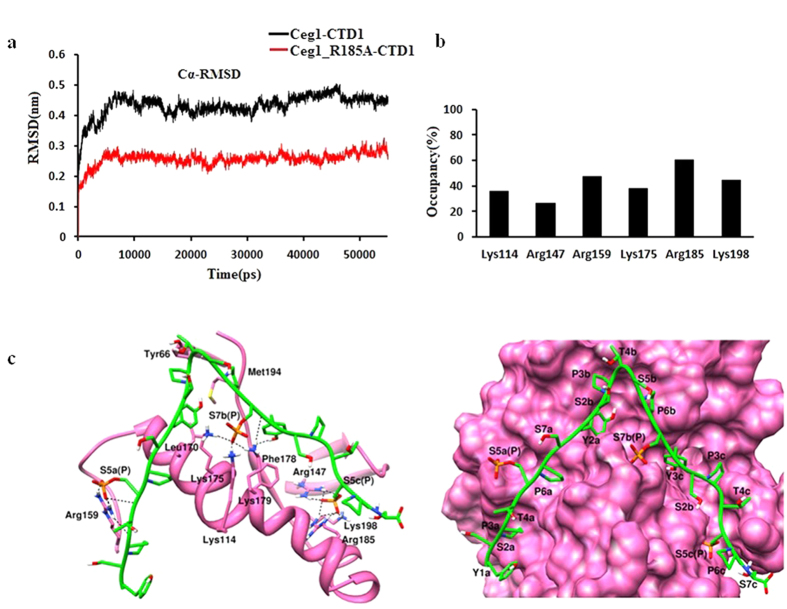
MD simulation of Ceg1-CTD1 and Ceg1_R185A_-CTD1. (**a**) Cα-RMSD of Ceg1-CTD1 and Ceg1_R185A_-CTD1 calculated over 55 ns MD simulation. (**b**) The occupancy of H-bonds formed between CTD1 and Ceg1. **(c)** Left panel shows interaction of CTD1 with Ceg1 and the right panel shows the conformation of CTD1 over the surface of Ceg1.

**Figure 8:**
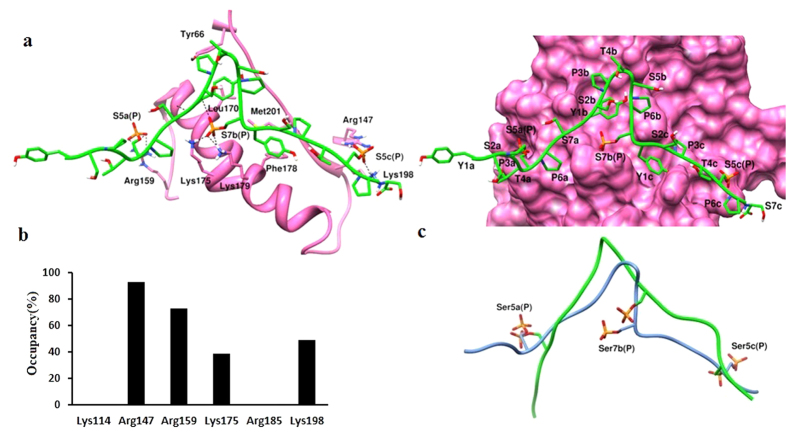
The mutation R185A alters the interaction of CTD1 with Ceg1. (**a**) Left panel shows an interaction between CTD1 and Ceg1_R185A_. The right panel shows the conformation of CTD1 over the surface of Ceg1_R185A_. (**b**) H-bond occupancy analysis of Ceg1_R185A_-CTD1 interaction. (**c**) The aligned conformations of CTD1 with Ceg1 (green) and Ceg1_R185A_ (blue). For the interaction analysis, the last frame obtained after 55 ns MD simulation was used.

Since CTD1 showed better interaction with Ceg1, we further carried out a separate long (55ns) MD simulation studies of CTD1 bound to NT domain of Ceg1 (1–242) (Fig. 7a). Cα-RMSD trajectory showed that the Ceg1- CTD1 complex is stable during the MD simulation. Here, with increase in the simulation length, CTD1 appeared to interact more strongly with the Ceg1. The phosphorylated Ser7b showed extensive interaction with Lys114 and Lys179 (Fig. 7b,c). The above results suggest an interaction between Ceg1 and CTD1 with more specificity for 5a7b5c (YSPTS_P_PS-YSPTSPS_P_-YSPTS_P_PS) manner. Using MD simulation studies, we have also investigated the effect of Ceg1_R185A_ and Ceg1_K198A_ mutations on CTD1 binding. Since the cells bearing Ceg1_R185A_ mutation did not grow optimally, we speculated that the loss of interaction between phosphorylated Ser_5_c and Ceg1_R185A_ will have significant impact on CTD1 binding. In case of Ceg1R_185A_, CTD1 adopts a different conformation on the CID surface as compared to Ceg1 (Fig. 6b), and phophorylated Ser_5_ of third heptad showed interaction with Arg147 and Lys198. In addition, phosphorylated Ser_5_ of first heptad and Ser_7_ of second heptad showed interaction with Arg159 and Lys175 respectively. The hydrophobic/hydrophilic interactions analysis shows a compromised hydrophobic interaction between Ceg1_R185A_ and CTD1. For Ceg1, Ceg1_R185A_ and Ceg1_K198A_, the areas of lipophilic surface matches with CTD1 were of 89.30 Å^2^, 16.52 Å^2^ and 63.73 Å^2^ respectively. In case of Ceg1_K198A_, CTD1 maintained the hydrophobic interaction and the loss of hydrophobic interaction for Ceg1_R185A_, appears to affect the CTD1 binding (Table 1). It has been reported that Tyr and Pro residues in CTD repeat are involved in hydrophobic interaction with the CID^9^ and hence the contribution of hydrophobic interaction in CTD-Ceg1 interaction cannot be ruled out. The binding of CTD1 with Ceg1_R185A_ was further investigated by carrying out a separate 55 ns MD simulation (Figs 7a and 8a). The CTD1 showed a similar interaction pattern as explained in the previous 15ns MD simulation (Fig. 8b). We observed that in both 15 ns and 55 ns of MD simulation, CTD1 adopt different conformations in Ceg1-CTD1 and Ceg1_R185A_-CTD1 (Fig. 8c). The calculations obtained from the PLATINUM and PDBePISA servers reveal that in case of Ceg1_R185A_, there is a significant decrease in the binding affinity for CTD1 (Tables 1 and 2).

## Discussion

The presence of Ser_7_ at the most degenerate position in CTD heptads (appearing 26/52 in human, 7/24 in *drosophila*, 19/26 in yeast) suggests its specialized function^1^,^2^. This is supported by the fact that the presence of only consensus CTD repeats (52 repeats with YSPTSPS) in mammals shows reduced growth compared to the wild type cells^21^. The current knowledge of CTD based transcription progression of mRNA is mostly based on the phosphorylation at Ser_2_ and Ser_5_. Lately identified Ser_7P_ and the presence of this mark as observed by ChIP and ChIP chip signals at 5′ end, middle and 3′ end of the protein coding genes, makes the whole transcription cycle more complicated and dynamic. It further suggests that the role of Ser_7P_ is not limited to the snRNA transcription only. In addition to the probable specialized function of this mark, the combinatorial possibility of the differential phosphorylation and its subsequent function also cannot be ruled out. As evident, the integrator recruitment to CTD was found to be influenced by Ser_2P_ + Ser_7P_ double mark during snRNA transcription^22^. Hence, it is too preliminary to conclude that the different transcription regulators bind due to specifically phosphorylated Ser_2_ and/or Ser_5_ only.

The non-homologous Ser_5_ mutations of CTD are synthetically lethal. In *Saccharomyces cerevisiae*, the Cet1-Ceg1 complex is thought to be recruited to Ser_5P_ of CTD^3^,^4^,^6^. In the process of establishing the interaction between Ceg1 and CTD, a role of Ser_7_ has always been overlooked due to the fact that this phosphorylation was not known at the time of studies being carried out on the Ceg1-CTD interaction and its subsequent role in the mRNA capping or other similar studies. The *in vitro* pull down, and yeast two hybrid analysis with mutant CTD suggests the interaction of Ceg1 with CTD. The observed effect of Ser_7_ mutation on the growth and mRNA capping suggests its role beyond snRNA transcription and of biological significance. The insignificant change in the mRNA capping due to Ser_7_ mutation suggests the role as one among many which could influence the mRNA transcription. The major possibilities are of its role being as a place keeper for other CTD binding proteins which inadvertently affects different process of transcription^19^. The structural superimpositions of Ceg1-CTD and Cgt1-CTD identifies key residues of budding yeast mRNA capping enzyme, having potential to interact with Ser_5_ and Ser_7_ of CTD. The absence of significant interaction between Lys175 of Ceg1 and CTD is attributed for the strong preferential binding with Ser_5P_ of alternate CTD heptad and hence the Ser_7P_ appears to act as a place keeper residue. The MD simulation studies of Ceg1 with a tri-heptad CTD phosphorylated as 5a7b5c (YSPTS_P_PS-YSPTSPS_P_-YSPTS_P_PS) manner supports the interaction of Arg159, Lys175 and Arg198 in the given manner. Our studies explore the role of Ser_7_ phosphorylation in mRNA transcription and also show a pattern of CTD phosphorylation not described before for the recruitment of mRNA capping enzyme.

## Methods

### Cloning, expression and purification of Ceg1 and mutants

*ceg1* gene was PCR amplified from the genomic DNA of *S. cerevisiae* (S288C) using primer pair 5′CTAGCTAGCATGGT ATTGGCA ATGGAAAGT AGAGTGGCA-3′ and 5′-ATAAGAATGCGGCCGCGTCAGACCAATCATCCTCAT CTA-3′. PCR conditions used were: 94 °C-5 min; 94 °C-1 min; 61 °C-1 min, 72 °C-1 min (30 cycles); 72 °C-10 min. The amplified fragments were digested with NheI and NotI and then ligated into the pET-21d (+) vector (Novagen) cut with the same enzymes. Competent DH5-α cells were transformed with the plasmid constructs and screened for positive clones. The mutants Ceg1_R159A_, Ceg1_K175A_, Ceg1_R185A_ and Ceg1_K198A_ were generated from the above construct using the GeneTailor™ Site-Directed Mutagenesis System (Invitrogen) and the mutagenic primer pairs ATCAACGGTGCGTGTCTCACACAATCACCA/GTGAGACACGCACCGTTGATAGCAAGACA,CACCTTGGAGCGGATTTTTTAAACCATAC/AAAAAATTCCGCTCCAAGGTGGGCTAGTCT,TTCGATTTAGCGGCAGCGTACCCTAATCGT/GTACGCTGCCGCTAAATCGAAGTATGGTTT,TTTCCGTTCGCGATTTCCATGAAACATATG/CATGGAAATCGCGAACGGAAAAGTAGTACA respectively. The conditions used for amplification were same as specified for use with Platinum *Pfx* DNA polymerase (Invitrogen). The mutants (Ceg1_R159A_, Ceg1_K175A_, Ceg1_R185A_ and Ceg1_K198A_) for over expression were cloned in pET-21d (+). Two hybrid plasmids pGBDU-C1 and pGAD-C1 code for the DNA binding (GBD) and transcriptional activation (GAD) domains of Gal4p respectively, and the construction of GBD-Ceg1, GAD-S2S5S7, GAD-A2S5S7 and GAD S2A5S7 have been described previously^23^,^24^. The mutants for the two hybrid analysis (GBD-Ceg1_R159A_, GBD-Ceg1_K175A_, GBD-Ceg1_R185A_ and GBD-Ceg1_K198A_) were cloned using TCCCCCGGGATGGTATTGGCAATGGAAAGTAGAGTGGCA/ GAAGATCTCG GCCGCG TCAGACCAATCATCCTCATCTA primer pairs. Sequences containing the mutated CTD repeats (14 heptads), S2S5A7, A2S5A7 and S2A5A7 were custom synthesized from IDT and cloned into pGAD-C1 vector as described previously^24^. The DNA sequencing of all the amplified genes confirmed the sequence homogeneity.

The BL21 (DE3) expressing Ceg1 or its mutant (all his-tagged) was inoculated into 500 ml of LB broth having ampicillin (100 μg/ml) and allowed to grow at 37 °C until A_600_ of 0.6 was achieved. The culture was then induced with 0.5 mM IPTG and incubated further at 37 °C for 4 hours. The cells were harvested and the resultant pellet was resuspended in lysis buffer containing 50 mM Tris Cl (pH 8), 100 mM NaCl and 2 mM PMSF and disrupted using a probe-type ultrasonicator followed by high speed centrifugation for 30 min at 4 °C. The supernatant was loaded onto the Ni NTA column, washed and eluted using 300 mM imidazole. The GST-CTD was purified and kinase assay was performed as described previously^8^,^25^.

### Fluorescence anisotropy

Measurements were carried out in a fluorescence spectrometer in T-configuration (Perkin Elmer LS50b) in buffer (25 mM HEPES pH-8, 100 mM NaCl, 1 mM EDTA, 1 mM DTT) at 25 °C as described previously^26^. For binding experiments, Ceg1 or mutants were titrated into a reaction mixture containing buffer supplemented with 2 μM of FAM-CTD-Ser_5P_ (YSPTS_P_PS-YSPTS_P_PS-YSPTS_P_PS). Data were fitted to the cubic equation applying nonlinear regression one site total binding mode as described in GraphPad Prism 5.

### Pull down assay

The GST-CTD was incubated for 4 hours at 4 °C with equilibrated glutathione beads in 20 mM HEPES-KOH (pH-7.3), 15 mM magnesium acetate, 100 mM potassium acetate, 1 mM DTT, 2.5 mM EGTA and 10% glycerol. After washing, the GST-CTD was phosphorylated by Kin28^25^, washed and incubated with Ceg1 or its mutants for overnight at 4 °C. The reaction mixture was extensively washed to remove the unbound proteins, before elution with reduced glutathione. In case of mutant analysis, after pull down assay the proteins were transferred onto nitrocellulose membrane and Ceg1 detected using anti his antibody.

### MD simulation

MD simulation studies were carried out with the help of Gromacs 4.5.5^27^. The Cgt1-CTD co-crystal structure was used to generate the 3D-models of CTD with different phosphorylation patterns (14). The Biopolymer module of SYBYLX-2.0 was used to generate 21 amino acid CTD heptad and subsequent phosphorylation of Ser residues using the phosphorylate tool^28^. To preserve the crystal conformation of CTD, the energy minimization on the generated peptides were not carried out. The generated CTD repeats were then positioned on the mapped CTD interaction interface of Ceg1 using the structural alignment method available in UCSF Chimera1.6^29^. Each of Ceg1-CTD complexes was subjected to MD simulation under Gromos43a1 forcefield^30^. The SPC water model was used to solvate the complexes in the periodic cubic box. Na^+^ and Cl^−^ ions were added to neutralize the systems at a concentration of 0.1 M. After minimization of solvated systems, NVT and NPT equilibration were carried out for 500 ps and 1 ns respectively. The temperature of the system was maintained at 300 K. Finally, systems were subjected to 15 ns production simulation.

### Yeast two hybrid

Two-hybrid plasmids pGBDU-C1and pGAD-C1 code for the DNA binding (GBD) and transcriptional activation (GAD) domains of Gal4p, respectively (15). Two hybrid plasmids pGAD-S2S5A7(14 repeats), pGAD-A2S5A7(14 repeats), pGAD-S2A5A7 (14 repeats), were constructed by fusing mutant DNA sequences coding for the CTD of Rpb1 to DNA coding for transcriptional activation domain of Gal4p in pGAD-C1. Sequences containing the mutated CTD repeats were custom synthesized from IDT. The assay was performed by transforming the strain PJ69-4A with different GAD plasmids to the Ceg1-GBD and their growth was assayed on synthetic drop-out medium containing appropriate amino acids supplemented with 2% glucose. Growth on lacking medium is due to the expression of the reporter gene (HIS3) by the interaction of the two hybrid fusion proteins fused upstream of the DNA binding (GBD) and transcriptional activation (GAD) domains of Gal4p. A 10 μl aliquot of serial 10-fold dilutions were spotted on sc-ura-leu-his plates and photographed after incubation at 30 °C for 36 hours.

### RNA Immunoprecipitation

The experiment was performed as described previously^31^. Briefly, yeast strain with consensus and with Ser7A mutant (14 repeats) were grown at 30 °C and harvested at mid log phase for RNA isolation. Protein A/G plus agarose beads were washed three times with 1.5 ml of buffer IPP150 (150 mM NaCl/0.1% Nonidet P40/10 mM Tris, pH 8.0) and three times with 1.25 ml of buffer IPP500 (500 mM NaCl/ 0.1% Nonidet P40/10 mM Tris, pH 8.0) and resuspended in 100 μl of buffer IPP500/reaction. H20 antibody (5 μg per reaction) was added and rotated at 4 °C overnight on a tube rotator to couple the beads to the antibody. H20 antibody recognizes the 2,2,7trimethylguanosine (m3G) containing cap structure. After coupling, the beads were washed three times with IPP150 and resuspended in the IP reaction mix (5 μg of total RNA,DTT, RiboLock RNase Inhibitor and IPP150 buffer up to a total volume of 200 μl). Mock IP reaction with no antibody served as nonspecific binding control. IP reactions were rotated at 4 °C overnight. The beads were washed five times in 1 ml of cold IPP150 containing 2.5 mM DTT and resuspended in 200 μl of Proteinase K solution and put on a tube rotator at 37 °C for 30 min to recover the RNA from the pellet. Then 200 μl of IPP150, 20 μl of glycogen (10 mg/ml) and 400 μl of acid phenol:chloroform were added to each sample. RNA was extracted by vortexing for 15 seconds and spinning for 5 min at maximum speed and room temperature. After ethanol precipitation, the RNA pellets were resuspended in DEPC treated water. mRNA capping experiments was performed exactly as described previously^32^.

## Additional Information

**How to cite this article**: Bharati, A. P. *et al*. The mRNA capping enzyme of *Saccharomyces cerevisiae* has dual specificity to interact with CTD of RNA Polymerase II. *Sci. Rep.* **6**, 31294; doi: 10.1038/srep31294 (2016).

## Figures and Tables

**Table 1:** The hydrophobic/hydrophilic interaction analysis using PLATIMUM tool (www.model.nmr.ru/platinum).

Ligand	#H-bonds	S(L/L)	S(H/H)	S_buried_	S_total_	Match^1^	Match^2^
WtCeg1-CTD1	(9.57)	(99.05)	(523.33)	(861.38)	(1878.89)	(0.3360)	(0.3195)
	9.42	89.30	391.13	650.18	1754.01	0.2739	0.3305
Ceg1R185A-CTD1	(8.11)	**(83.73)**	(329.99)	(517.40)	(1818.31)	(**0.2275**)	(0.3360)
	10.12	**16.52**	409.36	725.68	1827.38	**0.2331**	(0.0702)
Ceg1K198A-CTD1	8.09	63.73	412.40	675.11	1808.20	0.2461	0.2461

S(L/L), S(H/H), S_buried_, S_total_, Match^1^, Match^2^ represent an area of lipophilic match surface (Å^2^), hydrophilic match surface (Å^2^), ligand buried surface (Å^2^), total surface area (Å^2^), fraction of matching total surface (Å^2^) and fraction of matching hydrophobic surface (Å^2^) respectively. Values in brackets are obtained after analysis of last frame obtained after 55 ns MD simulation.

**Table 2:** Interface analysis using PDBePISA server (www.ebi.ac.uk/pdbe/pisa/).

Ligand	N_HB_	Interface area (Å^2^)	Δ^i^G (Kcal/mol)
Ceg1-5a7b5c	15	1256.6	−24.2
Ceg1_R185A_-5a7b5c	**9**	**720.8**	**−14.7**
